# Misdirected Patients in Orthopedic Outpatient Clinics: A Retrospective Four Years Data Analysis (23435 Patients)

**DOI:** 10.7759/cureus.6526

**Published:** 2019-12-31

**Authors:** Syed Muhammad Azfar, Manal Abdulaziz Murad, Syeda Azim, Mukhtiar Baig

**Affiliations:** 1 Orthopedics, Liaquat College of Medicine and Dentistry, Karachi, PAK; 2 Family Medicine, King Abdulaziz University, Jeddah, SAU; 3 Medical Education and Simulation, Dow University of Health Sciences, Karachi, PAK; 4 Medical Education and Simulation, King Abdulaziz University, Jeddah, SAU

**Keywords:** orthopedic triage, orthopedic opd cases, misdirect patients

## Abstract

Objective

To identify the frequency of misdirected patients in orthopedic outpatient clinics.

Methodology

This was a retrospective study done in a private hospital of Jeddah. Computer records of patients attending the orthopedic outpatient department (OPD) during the period of 2013-2017 were collected. Data were analysed using IBM SPSS version 23 (IBM Corp, Armonk, NY). Descriptive statistics are presented as frequency and percentages.

Results

Out of the 23435 cases, 6944 (29.6%) cases should not be seen primarily in orthopedic clinic, 13638 (58.2%) were the cases that may or may not be seen primarily in orthopedic clinic, whereas, there were 2853 (12.2%) who must be seen mainly by orthopedic specialist.

Conclusion

This study revealed that a huge number of patients who visited orthopedic OPD does not need primarily orthopedic consultation. So, it is recommended to direct the patients to the right specialist in outpatient clinics to avoid the unnecessary burden on orthopedic clinics. The triage of referrals or walk-in patients may help to prevent this issue.

## Introduction

Orthopedic outpatient department (OPD) waiting lists are usually very long, and a large number of patients visit orthopedic OPD every day. But, most of the patients visiting orthopedic OPD do not need to be seen primarily by an orthopedic specialist. Their conditions can be seen and managed by physiotherapists, rheumatologists or family medicine physicians. These medical specializations are so much overlapped with each other that it is often very difficult for the patients to decide themselves that which type of doctor should they visit for their medical condition [1]. Orthopedic specialist doctors treat patients with joint and bone problems such as rheumatologists and physiotherapists. Orthopedic surgeons mainly deal with bone and joint diseases and injuries requiring surgical treatment, such as osteoarthritis and traumatic injuries to extremities and spine, etc. [2]. While, rheumatologists are internal medicine physicians who focus on autoimmune conditions and the non-surgical treatment of diseases, such as different types of arthritis, where medications alone or with physical therapy can provide the proper management [1]. While a physiotherapist is a medical professional who can treat the general joint pain by physical therapy [3].

It is commonly observed that due to lack of triage, many patients with non-traumatic joint or muscle conditions like generalized body aches, night pains, rheumatoid arthritis, nonspecific neck and back pain which are not requiring any surgical interventions often directed firstly to the orthopedic clinic [1,4]. It increases the burden of orthopedic clinics, especially, in public sector hospitals [2]. This reduces the efficacy of the orthopedic clinic and may affect the quality of care of patients. The triage of patients may help to prevent this issue, but this system is dependent on accurate and thorough information being provided by patients or in referral letters [5].

There is a scarcity of literature on this topic, so we decided to review our orthopedic clinical data retrospectively in this regard. Our study will add this new aspect to the scientific literature. This study aimed to identify the frequency of misdirected patients in orthopedic outpatient clinics retrospectively among 23435 patients who attended OPD from 2013-2017 at a secondary care hospital, Jeddah, Saudi Arabia.

## Materials and methods

This retrospective study was conducted in a private secondary care hospital located in Jeddah. Data were collected by reviewing computer records of patients who attended the orthopedics OPD between the period of 2013 and 2017. We divided our patients according to the diagnosis mentioned in their medical records. The duplicated patients (those who had visited the orthopedics OPD more than one time with the same diagnosis) were removed. This private hospital is in the centre of the city, and specialist orthopedic surgeons work in the OPD. This research is approved by the ethical committee. The personal identity of patients included in this study was kept confidential. Data were analysed using IBM SPSS version 23 (IBM Corp, Armonk, NY). Descriptive statistics are presented as frequency and percentages.

## Results

Out of the 23435 cases, 6944 (29.6%) were found not primarily requiring orthopedic specialist consultation (group C). Whereas, 13638 (58.2%) were the cases that may or may not be firstly seen in the orthopedic specialist clinic (group B). Furthermore, there were only 2853 (12.2%) patients identified who were primarily requiring orthopedic specialist consultation (group A).

Among the total 19337 male subjects, 2383 (12.3%), 5325 (27.5%), 11629 (60.2%) cases were in group A, B and C, respectively (p < .001). Of the total 4098 female subjects, 470 (11.5%), 1619 (39.5%), 2009 (49%) cases were in group A, B and C, respectively (p < .001).

Of the total 1756 subjects who belonged to the age group 1-18 years, 186 (10.6%), 429 (24.4%), and 1141 (65%) cases were in group A, B, and C, respectively (p < .001). Of the total 18123 subjects who belonged to the age group 19-50 years, 2229 (12.3%), 6374 (35.2%), and 9520 (52.5%) cases were in group A, B, and C, respectively (p < .001). Of the total of 3556 subjects who belonged to the age group >50 years, 438 (12.3%), 961 (27%) and 2157 (60.7%) cases were in group A, B, and C, respectively (p < .001) (Table 1).

**Table 1:** Gender and age-wise comparison of primarily orthopedic, not primarily orthopedic and problems may or may not be seen in Orthopedic Outpatient Department (OPD).

	Total	Group A (Primarily orthopedic) N (%)	Group B (Not primarily orthopedic) N (%)	Group C (May or may not be seen by orthopedic) N (%)	P-value
Cases	23435 (100)	2853 (12.2)	6944 (29.6)	13638 (58.2)	<0.001
Gender					
Male	19337 (82.5)	2383 (12.3)	5325 (27.5)	11629 (60.2)	<0.001
Female	4098 (17.5)	470 (11.5)	1619 (39.5)	2009 (49)	<0.001
Age					
1-18 years	1756 (7.5)	186 (10.6)	429 (24.4)	1141 (65)	<0.001
19-50 years	18123 (77.3)	2229 (12.3)	6374 (35.2)	9520 (52.5)	<0.001
>50 years	3556 (15.2)	438 (12.3)	961 (27)	2157 (60.7)	<0.001

Out of the 23435 cases, 4289 (18.3%) were suffering from tendinopathies/enthesopathies (plantar fasciitis, shoulder tendinosis, tennis elbow, impingement syndrome, and others), all of these should not primarily be seen by an orthopedic specialist. Among all subjects, 2486 (10.6%) were suffering from different types of arthritis including gout, rheumatoid arthritis, were not primarily orthopedic problems while osteoarthritis knee may or may not be seen in orthopedic OPD depending on the severity of the problem. A total of 1750 (7.4%) were suffering from nonspecific body-aches such as myalgia, generalized body-aches, polyarthralgia, and all these cases are not primarily orthopedic cases.

There were 1496 (6.4%) patients suffering from traumatic sprains, including foot, wrist, elbow, ankle, sternoclavicular sprains, which may or may not be seen in orthopedic OPD. Among all the neck pain cases, 1134 (4.8%), the neck muscle spasm, and parascapular muscle spasm were not primarily orthopedic cases (Table 2).

**Table 2:** Number of primarily orthopedic, not primarily orthopedic and problems may or may not be seen in the Orthopedic Outpatient Department (OPD) among several diagnoses.

Diagnosis N (%)	Group A (Primarily orthopedic) N (%)	Group B (Not primarily orthopedic) N (%)	Group C (May or may not be orthopedic) N (%)
Lower back pain N = 6089 (26.8)			
Mechanical low back pain			3898 (64)
Neuropathic low back pain			2087 (34.3)
Others		104 (1.7)	
Tendinopathies/Enthesopathies N = 4289 (18.3%)			
Plantar fasciitis		965 (22.4)	
Shoulder tendinosis		720 (16.7)	
Tennis elbow		490 (11.4)	
Impingement syndrome		387 (9)	
Metatarsalgia		348 (8.1)	
Others		1379 (32.2)	
Arthritis N = 2486 (10.6)			
Osteoarthritis knee			2296 (92)
Gout		129 (5.2)	
Rheumatoid arthritis		29 (1.2)	
Others		32 (1.3)	
Fractures 2567 (10.9)			
A. Small bone fractures 1353 (5.3)			
Phalanges	635 (46.9)		
Metacarpals & metatarsals	380 (28)		
Clavicle	145 (10.7)		
Calcaneus	166 (12.3)		
Others	27 (1.9)		
B. Long bones fracture 1214 (4.7)			
Distal radius	82 (6.7)		
Forearm (both bones)	312 (25.7)		
Humerus	185 (15.2)		
Tibia shaft fracture	258 (21.2)		
Hip fracture	77 (6.3)		
Femur shaft	103 (8.4)		
Others	197 (16.2)		
Soft tissue injuries 2334 (9.9)			
Contusions			1659 (71.1)
Crush injuries			118 (5)
Deep lacerations			107 (4.6)
Deep venous thrombosis		30 (1.3)	
Foreign body			39 (1.7)
Tendon injuries			157 (6.7)
Wound (Skin lacerations)			214 (9.2)
Abrasions			6 (.3)
Haematoma		4 (.2)	
Non-specific body-aches 1750 (7.4)			
Myalgia		1489 (85)	
Generalized body-aches		78 (4.5)	
Polyarthralgia		183 (10.5)	
Traumatic sprains 1496 (6.4)			
Foot sprains			417 (28)
Wrist sprains			206 (13.8)
Elbow sprains (Adult)			71 (4.7)
Ankle sprains			797 (53.3)
Sternoclavicular sprains			5 (.3)
Joints stiffness 414 (1.8)			
Frozen shoulder			343 (83)
Joint stiffness (Others)			71 (17)
Neck pain 1134 (4.8)			
Neck muscle spasm		329 (29)	
Cervical radiculopathy			457 (40.3)
Cervical spondylosis			167 (14.7)
Parascapular muscle spasm		181 (16)	
Infections 326 (1.42)			
Infected wounds			83 (25)
Osteomyelitis	24 (7.2)		
Cellulitis			94 (28.2)
Diabetic foot			123 (37)
Madura foot			2 (.6)
Septic arthritis	7 (2)		
Sports 218 (1)	218		
Nerve injuries 20 (0.08)			20
AVN 37 (0.16)	37		
Tumours 201 (0.85)			201
Metabolic 67 (0.3)		67 (.3)	
Total = 23435	2853 (12.2%)	6944 (29.6%)	13638 (58.2%)

## Discussion

Our study showed that during the period of 2013-2017, out of 23,435 patients who visited orthopedic outpatient clinics, 6944 (29.6%) patients did not need primarily orthopedic consultation while 13638 (58.2%) patients can be seen by an orthopedic specialist or by some other specialist. In contrast, only 2853 (12.2%) patients were in the group who primarily needed orthopedic consultation. This study found that a good number of patients who visited orthopedic surgery clinics did not primarily require orthopedic specialist consultation. Out of 52 diagnoses in patients visiting orthopedic OPD, only 15 diagnoses were those who must be seen by an orthopedic specialist, while 17 diagnoses should not be seen primarily by an orthopedic physician, whereas 20 diagnoses can be seen by an orthopedic specialist or by some other specialty. Speed and Crisp (2005) also found in their study that almost 40% of the referral patients came to the orthopedics OPD did not need to see an orthopedic specialist rather, they needed a visit to rheumatologist OPD. They further proposed that there should be an effective referral system that contains necessary information about patients to direct patients to the right specialist. Moreover, it needs signiﬁcant education and training for general practitioners [1]. It is also recommended that physiotherapy can be offered as a primary management option [6]. Similarly, improving the clinical skills of general practitioners in managing specific musculoskeletal problems is also advisable [7].

In the current era, health professionals are required to practice through great collaboration. As the number of patients with medical conditions like low back pain, neck pain, and other musculoskeletal conditions is continuously increasing, so referring them to trained physiotherapist or rheumatologist through proper triage system would prevent the backlog of waiting patients for a single specialist clinic [3]. There are many potential reasons for this issue; for example, lack of recognition of orthopedic disorders by OPD receptionists as most of the patients visited orthopedic surgeons as a walk-in patient. Whereas, if a doctor is referring to a patient, then there may be lacking in writing the right information in the referral letter [6]. Many researchers found that the triage system enhances and helps in filtering the patients referred from the general practitioners. But in a walk-in clinic, when a patient comes to the orthopedic clinic, triage can be done by reception staff to an alternate specialist as per need [6]. In our setup, most of the patients visit an orthopedic clinic as a walk-in patient, so in this case, reception or a nursing staff triage system might work. A ‘Triage Nurse’ is a registered trained nurse who can be posted in an outpatient department and is responsible for directing patients to the correct specialty [7].

In this study, we have found that 26% of the patients came with the complaint of mechanical lower back pain. Lower back pain (LBP) can be managed by rheumatologists, chiropractor or physiotherapists as well. Only a few cases of LBP would need an orthopedic consultation [8]. A study done in Australia showed that nearly two-thirds of patients with a non-urgent musculoskeletal condition were appropriately assessed and managed by an experienced, qualified physiotherapist [9]. Worldwide, LBP is the most prevalent and disabling musculoskeletal conditions in the community. It places enormous demands on primary care and hospital resources. This foremost musculoskeletal complaint is seen abundantly in both general practice as well as in hospital emergency departments [10,11]. Syed et al. recommended the need for separate back pain clinics to reduce the burden on the orthopedic clinics [12]. Moi et al. experimented with Backpain Assessment Clinic (BAC) which is an alternative pathway that provides patients with streamlined access to community-based expert assessment and spinal rehabilitation, physiotherapist and rheumatologist. The results of this study suggest that BAC is a potentially safe and cost-saving alternative model of care, and it also improves the orthopedic OPD effectiveness [13].

This research revealed that a good number of misdirected patients who visited orthopedic OPD were suffering from tendinopathies and enthesopathies. These can be seen by a rheumatology specialist. The surgical option for these conditions remains the last option after exhausting all nonoperative options [14]. The pure orthopedic cases like fractures and other conditions requiring surgeries in our study contribute about twelve percent of the total load of the clinic. This result reflects that these important cases did not get enough consultation time that they deserve to get, which may lead to patient dissatisfaction or missed diagnosis. Long waiting times, insufficient time with a specialist are the commonest complaints about the clinics [2]. Patients often complain that they had not been able to say all they wanted to the specialist, given that lots of the irrelevant appointments and referrals might be the main reasons for the avoidable liabilities in orthopedic clinics, and it is necessary to develop an effective triage system without compromising the quality of patient care [14].

Non-surgical musculoskeletal disorders pose a considerable burden on the orthopedic OPD. The patients with higher needs should be prioritized in orthopedic clinics, and there is a potential for increased morbidity and negligence. Therefore, we need to create ways to reduce the unnecessary burden on orthopedic clinics by establishing an effective triage system, pain management and back pain clinics, etc. that will ultimately reduce the unwanted load on the orthopedic OPD leading to improved patient care and satisfaction.

Limitations

The finding of this study cannot be generalized because we did convenience sampling. There is also a chance of observational bias, as a grouping of the diagnoses was done subjectively, which can vary on physicians and medical centers’ clinical practices. Furthermore, we did not include the data of patients whose information was incomplete, so it may not represent the actual data.

## Conclusions

In conclusion, this study revealed that a good number of patients who visited orthopedic clinics did not need to see orthopedic specialist primarily. Out of the 23435 cases, 6944 (29.6%) were not primarily orthopedic cases, 13638 (58.2%) were the cases that may or may not be seen in orthopedic OPD, while there were only 2853 (12.2%) primarily orthopedic cases. Thus, there were many patients who were misdirected towards orthopedic OPD. That is why there is a great need to direct the patients to the right specialist in the OPDs to avoid the unnecessary burden on orthopedic clinics, which will also save the patients’ precious time and money. The triage of referrals or walk-in patients may help to prevent this problem.

## References

[1] Speed CA, Crisp AJ (2005). Referrals to hospital-based rheumatology and orthopaedic services: seeking direction. Rheumatology.

[2] Roland MO, Porter RW, Matthews JG, Redden JF, Simonds GW, Bewley B (1991). Improving care: a study of orthopaedic outpatient referrals. BMJ.

[3] Brismée JM, Pape JL, Woodhouse LJ (2018). Reflections and future directions on extending physical therapist scope of practice to improve quality of care and preserve health care resources. Phys Ther.

[4] Napier C, McCormack RG, Hunt MA, Brooks-Hill A (2013). A physiotherapy triage service for orthopaedic surgery: an effective strategy for reducing wait times. Physiother Can.

[5] Burn D, Beeson E (2014). Orthopaedic triage: cost effectiveness, diagnostic/surgical and management rates. Clin Gov Int J.

[6] Morris J, Grimmer-Somers K, Kumar S (2011). Effectiveness of a physiotherapy-initiated telephone triage of orthopedic waitlist patients. Patient Relat Outcome Meas.

[7] George S, Read S, Westlake L, Williams B, Pritty P, Fraser-Moodie A (1993). Nurse triage in theory and in practice. Emerg Med J.

[8] Piccoliori G, Engl A, Gatterer D, Sessa E, in der Schmitten J, Abholz HH (2013). Management of low back pain in general practice - is it of acceptable quality: an observational study among 25 general practices in South Tyrol (Italy). BMC Fam Pract.

[9] Oldmeadow LB, Bedi HS, Burch HT, Smith JS, Leahy ES, Goldwasser M (2007). Experienced physiotherapists as gatekeepers to hospital orthopaedic outpatient care. Med J Aust.

[10] Pearse EO, Maclean A, Ricketts DM (2006). The extended scope physiotherapist in orthopaedic out-patients - an audit. Ann R Coll Surg Engl.

[11] Azfar SM, Murad MA, Azim SR, Baig M (2019). Frequency of and various factors associated with stress, anxiety, and depression among low back pain patients. Cureus.

[12] Syed MA, Azim SR, Baig M (2019). Frequency of orthopedic problems among patients attending an orthopedic outpatient department: a retrospective analysis of 23 495 cases. Ann Saudi Med.

[13] Moi JH, Phan U, de Gruchy A, Liew D, Yuen TI, Cunningham JE, Wicks IP (2018). Is establishing a specialist back pain assessment and management service in primary care a safe and effective model? Twelve-month results from the Back pain Assessment Clinic (BAC) prospective cohort pilot study. BMJ Open.

[14] Andres BM, Murrell GA (2008). Treatment of tendinopathy: what works, what does not, and what is on the horizon. Clin Orthop Relat Res.

